# Comparing the use of radiolabeled SSTR agonists and an SSTR antagonist in breast cancer: does the model choice influence the outcome?

**DOI:** 10.1186/s41181-017-0030-z

**Published:** 2017-09-29

**Authors:** Simone U. Dalm, Marion de Jong

**Affiliations:** Department of Radiology & Nuclear Medicine, Erasmus MC, Rotterdam, South Holland Netherlands

## To the Editor

With interest we read the recent publication of Dude et al. (2017) on the evaluation of somatostatin receptor (SSTR) agonists and an antagonist for SSTR-mediated imaging of breast cancer using positron emission tomography. In this study the authors compared 2 SSTR agonists (DOTA-Tyr^3^-octreotide and DOTA-Tyr^3^-octreotate) and the SSTR antagonist (NODAGA-JR11) in in vitro binding and saturation studies and in *in vivo* imaging and biodistribution studies. To our surprise their results demonstrated both agonists to have a more favorable receptor binding affinity and a better tumor uptake *in vivo*, whereas the saturation assay resulted in more binding sites for ^67/nat^Ga-DOTA-Tyr^3^-octreotide on the used breast cancer cell line (ZR75–1) than ^nat^Ga-NODAGA-JR11 and ^67/nat^Ga-DOTA-Tyr^3^-octreotate.

The reported results are in contrast with previously published studies comparing radiolabeled DOTA-Tyr^3^-octreotate and DOTA-JR11 in various tumor models (Dalm et al., 2016; Nicolas et al., 2017; Reubi et al., 2017; Wild et al., 2014), including our recent publication on the use of SSTR agonists and antagonists for targeting of breast cancer (Dalm et al., 2017). The main explanation given by the authors for the contradicting results is the use of an endogenously SSTR expressing breast cancer cell line, ZR75–1, in contrast to transfected cell lines, cell lines of other cancer types and non-cancerous cell lines used in earlier studies evaluating SSTR-targeting radiotracers.

Concerning the above mentioned explanation of the authors, we have the following remarks:

- First, some of the non-cancerous cell lines and cell lines of other cancer types used in previous studies also have endogenous SSTR expression. One example is our previously published study in which we reported better therapeutic efficacy with ^177^Lu-DOTA-JR11 compared to ^177^Lu-DOTA-Tyr^3^-octreotate in a xenograft model generated with the human small cell lung cancer cell line, H69 (Dalm et al., 2016).
- Furthermore, previously published studies comparing the use of radiolabeled JR11 and radiolabeled DOTA-Tyr^3^-octreotate or DOTA-Tyr^3^-octreotide were not only performed preclinically in tumor models, but also clinically in patients with neuroendocrine tumors. In the latter mentioned study published by Wild et al. (2014) ^177^Lu-DOTA-JR11 tumor uptake was superior to that of ^177^Lu-DOTA-Tyr^3^-octreotate. Although this study was not performed in breast cancer patients, it again demonstrates superiority of the SSTR antagonist vs the agonist in tumors that have endogenous SSTR expression.
- Concerning breast cancer, Reubi et al. (2017) demonstrated that binding of ^125^I–DOTA-JR11 to human breast cancer tissue was much higher than that of ^125^I–DOTA-Tyr^3^-octreotide. We also demonstrated higher binding of ^111^In-DOTA-JR11 vs ^111^In-DOTA-Tyr^3^-octreotate to 40 human breast cancer tissue samples (Dalm et al., 2017). Furthermore, in the same study we also showed higher *in vivo* tumor uptake of ^177^Lu-DOTA-JR11 vs ^177^Lu-DOTA-Tyr^3^-octreotate in an estrogen receptor positive patient derived breast cancer mouse model with endogenous SSTR expression.

Differences between the study of Dude et al. (2017) and our previous study (Dalm et al., 2017) include the use of different radionuclides and application of DOTA-JR11 instead of NODAGA-JR11. The authors chose NODAGA-JR11 because DOTA-JR11 has a lower receptor affinity when labeled with ^68^Ga. Similar to ^111^In-DOTA-JR11, ^177^Lu-DOTA-JR11 and ^177^Lu-DOTA-Tyr^3^-octreotate, ^68^Ga-NODAGA-JR11, ^68^Ga-DOTA-Tyr^3^-octreotate and ^68^Ga-DOTA-Tyr^3^-octreotide have comparable receptor affinity (Fani et al., 2012; Reubi et al., 2000).

Aspects concerning the methodology that to our opinion might influence the results when comparing different radiotracers, include:

- The use of different peptide amounts as also addressed by the authors in the discussion. The peptide amount of ^68^Ga-DOTA-Tyr^3^-octreotate used in the study was twice as high as the peptide amount of ^68^Ga-NODAGA-JR11. Although the authors mention that previous studies showed that within a range of 10–60 pmol tumor uptake of ^111^In-DOTA-Tyr^3^-octreotide is >80% of the maximum in rats (de Jong et al., 1999), this might be different in the model currently applied and this needs to be determined for the other radiotracers as well.
- The use of different peptide amounts for imaging and biodistribution studies.
- The use of an agonist (SRIF-28) in the competition assay to determine the binding affinity of the tracers. This would only be correct if the antagonist and the agonist have the same binding site, which is unclear.
- Also, in the study by Dude et al. (2017) imaging and biodistribution studies were performed at early time points (55 min and 60 min p.i., respectively), presumably because of the short half-life of ^68^Ga. However, in another study by Nicolas et al. (2017) it was reported that optimal tumor uptake of ^177^Lu-DOTA-JR11 and ^177^Lu-DOTA-Tyr^3^-octreotate was reached at 4 h p.i. as determined by biodistribution studies. Although there might be a difference in optimal tumor uptake when the same tracer is labeled with different radionuclides, the time point at which the imaging and biodistribution studies were performed might have contributed to the contradictory findings reported by in the study by Dude et al. (2017).

**Fig. 1:**
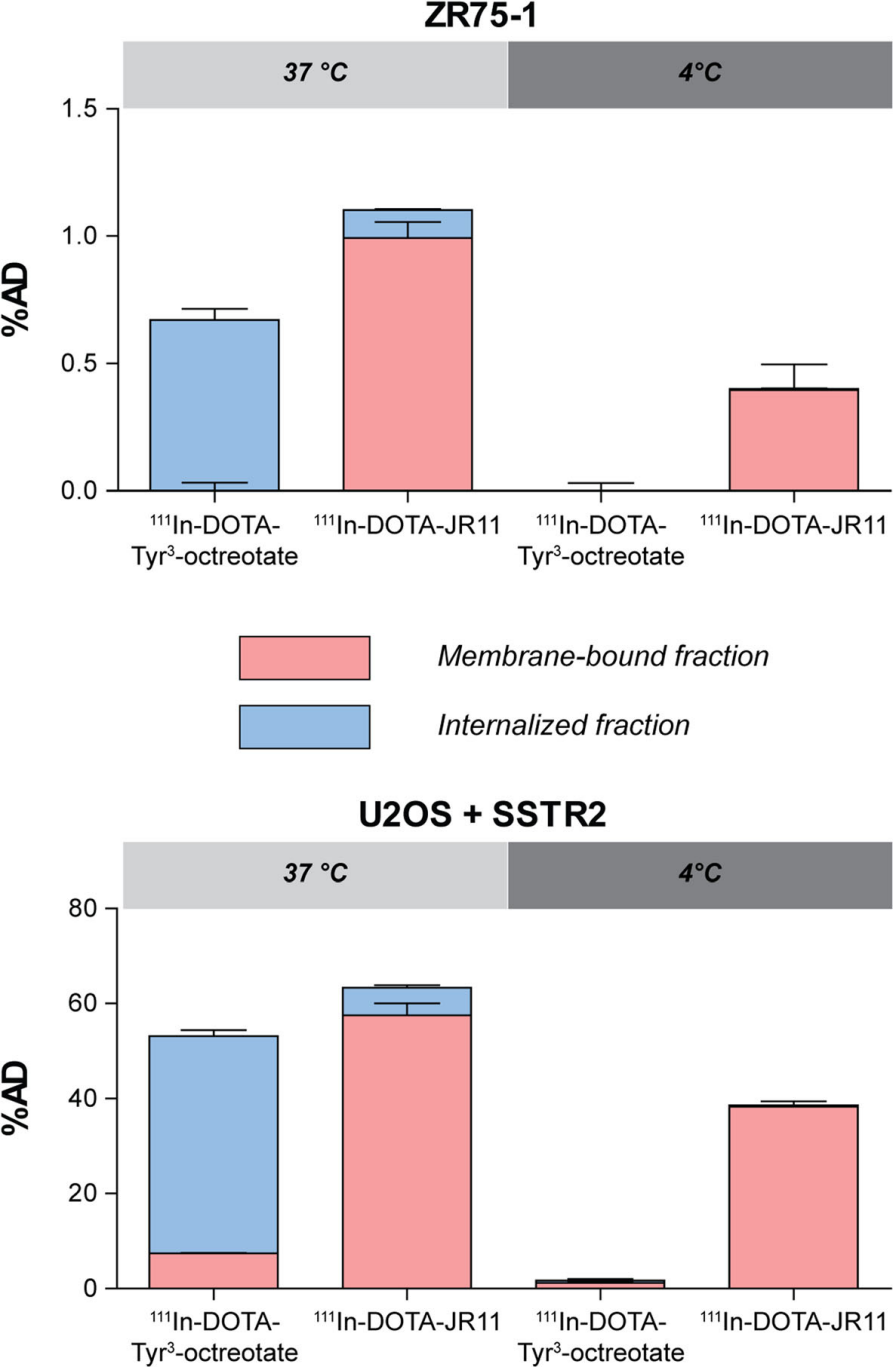
Binding and internalization of ^111^In-DOTA-Tyr^3^-octreotate and ^111^In-DOTA-JR11 to the endougenously SSTR expressing cell line ZR75–1 and the SSTR2 transfected cell line U2OS + SSTR2. Cells were incubated for 1 h at 37 °C or 4 °C with 10^−9^ M of the agonist or the antagonist. The bars represent the percentage added dose (%AD) bound and/or internalized (in)to the cells. The %AD is corrected for unspecific binding determined by blocking studies in which 10^−6^ M of the unlabeled agonist or antagonist was added

We recently compared binding of ^111^In-DOTA-JR11 and ^111^In-DOTA-Tyr^3^-octreotate to ZR75–1 and U2OS + SSTR2 (the latter is a human osteosarcoma cell line transfected with the SSTR2 receptor) in an internalization assay to investigate differences in SSTR agonist and antagonist binding to cell lines with endogenous and exogenous SSTR expression. The used method can be found in our previous paper (Dalm et al., 2016). Our results demonstrated that ^111^In-DOTA-JR11 is superior to ^111^In-DOTA-Tyr^3^-octreotate (even though the agonist is internalized) when applied for targeting of an endogenous SSTR-expressing cell line (ZR75–1) as well as for targeting the SSTR2 transfected cell line (Fig. 1).

Based on the above we conclude that endogenous SSTR expression of the model used in the study by Dude et al. (2017) does not explain the contradictory results obtained in this study. Multiple experiments in their study had a similar outcome and additional experiments are needed to determine what the reason is for these findings. However, in line with previous studies from our and other groups, the SSTR antagonist JR11 clearly shows superiority to the SSTR agonist octreotate for targeting breast cancer, also in the endogenously SSTR2 expressing breast cancer cell line ZR75–1.
